# Correction to “Secondary Sclerosing Cholangitis in a Critically Ill Burn Patient: A Case Report and Review of the Literature”

**DOI:** 10.1002/ccr3.72061

**Published:** 2026-02-12

A. N. Siniora, O. Marouf, R. R. Arekat, et al. “Secondary Sclerosing Cholangitis in a Critically Ill Burn Patient: A Case Report and Review of the Literature,” *Clinical Case Reports* 13, no. 12 (2025): e71549.

The ordering of the authors and their contributions was not described appropriately due to miscommunication between authors.

The ordering and contributions in the original text were

**Amanda N. Siniora:** resources, writing – original draft. **Omar Marouf:** writing – original draft. **Rasha R. Arekat:** resources. **Fatima A. Abuhilal:** methodology. **Mohammad I. Salah:** resources. **Mohammad AbuBaha:** visualization. **Hossam Salameh:** visualization, writing – review and editing. **Hatem M. Taha:** project administration.

The right ordering and contributions would be

**Amanda N. Siniora:** resources, writing – original draft. **Fatima A. Abuhilal:** methodology, writing – original draft. **Rasha R. Arekat:** resources, writing – original draft. **Mohammad I. Salah:** methodology, writing – original draft. **Omar Marouf:** resources. **Mohammad AbuBaha:** visualization. **Hossam Salameh:** visualization, writing – review and editing. **Hatem M. Taha:** project administration.

We apologize for this error.

